# Mycotoxins in Wheat Flours Marketed in Shanghai, China: Occurrence and Dietary Risk Assessment

**DOI:** 10.3390/toxins14110748

**Published:** 2022-10-31

**Authors:** Haiyan Zhou, Anqi Xu, Meichen Liu, Zheng Yan, Luxin Qin, Hong Liu, Aibo Wu, Na Liu

**Affiliations:** 1 SIBS-UGENT-SJTU Joint Laboratory of Mycotoxin Research, CAS Key Laboratory of Nutrition, Metabolism and Food Safety, Shanghai Institute of Nutrition and Health, University of Chinese Academy of Sciences, Chinese Academy of Sciences, Shanghai 200030, China; 2 Shanghai Municipal Center for Disease Control and Prevention, Shanghai 200336, China

**Keywords:** wheat flours, mycotoxins, contamination characteristics, risk assessment

## Abstract

The risk of exposure to mycotoxins through the consumption of wheat flours has long been a concern. A total of 299 wheat flours marketed in Shanghai Province of China were surveyed and analyzed for the co-occurrence of 13 mycotoxins through an ultra-high performance liquid chromatography-tandem mass spectrometry (UPLC-MS/MS) method. The detection rates of mycotoxins in wheat flours ranged from 0.7~74.9% and their average contamination levels in wheat flours (0.2~57.6 µg kg^−1^) were almost lower than the existing regulations in cereals. However, their co-contamination rate was as high as 98.1%, especially Fusarium and Alternaria mycotoxins. Comparative analysis of different types of wheat flours showed that the average contamination levels in refined wheat flours with low-gluten were lower. Based on these contamination data and the existing consumption data of Shanghai residents, point evaluation and the Monte Carlo assessment model were used to preliminarily evaluate the potential dietary exposure risk. The probable daily intakes of almost all mycotoxins, except for alternariol, were under the health-based guidance values for 90% of different consumer groups. Health risks of dietary exposure to alternariol should be a concern and further studied in conjunction with an internal exposure assessment.

## 1. Introduction

Wheat flour, which is primary processing food from wheat, is widely used to produce staple foods (breads, biscuits, cakes, pastries, pasta, and noodles). It can be divided into whole wheat flour and refined wheat flour for their differences in processing technology. Whole wheat flour includes the bran and germ, in addition to the basic endosperm, which are important sources of dietary fibers, vitamins, minerals, or healthy phytochemicals. The sales of whole cereal foods exceeded 23.68 billion dollars in China in 2015 [1]. It is also classified as low-gluten, medium-gluten, and high-gluten wheat flour based on protein content. This quality difference will affect the viscoelasticity and rheology of the dough, resulting in the chewiness and gumminess of its derived foods. Recently, organic wheat flours from special agricultural practice have also received more and more attention [2]. Differences in the choice of various types of wheat flours depend on various requirements in terms of food composition and quality for consumers.

Due to differences in climates, resistance, and other factors [3], organic or conventional wheat is still susceptible to mycotoxin contamination by toxigenic fungi, such as *Aspergillus* spp., *Fusarium* spp., *Penicillium* spp., and *Alternaria* spp., especially for their outer layer [4]. Preexisting mycotoxins in wheat grain with spatial localization [5] cannot be destroyed or eliminated by milling, but can be redistributed in different milling fractions, such as germ, bran, grits, or flour [6,7]. Multiple factors mentioned above have confounding effects on differences in mycotoxin concentrations in wheat flours [2,8]. Therefore, wheat flours may be contaminated by individual or multiple mycotoxins concurrently for some adverse factors during the whole industrial chain from agriculture practices to the table [9,10]. Mycotoxin contamination of wheat flour in various markets has also been reported continuously [11,12]. Mycotoxins in terms of deoxynivalenol (DON), ochratoxin A (OTA), zearalenone (ZEN), fusarenon-X (FUS-X), or neosolaniol (NEO) were detected in wheat and wheat flours from China [13,14,15,16]. DON was frequently detected in wheat flours marked in Hungary and the metabolic forms of DON were also found in spelt or durum flour [17]. OTA-contaminated wheat flours of 8% and 11.5% in Lebanon and Poland were also reported [18,19]. Fumonisins (FBs) were also common in wheat-based foods [10]. Recently, more focus has been placed on Alternaria mycotoxins (ATs) with the detection in wheat flour samples, which may be related to growth capacity at low temperature for *Alternaria* spp. [20,21]. The contamination rates of DON, alternariol (AOH), tenuazonic acid (TEA) and FB_1_ in 54 wheat flour samples from China were 90.7%, 16.7%, 3.7% and 9.3%, respectively [22]. Considering the public health concerns arising from their acute or chronic toxicity, the maximum levels of DON, ZEN, FB_1_, and OTA have already been proposed, not only in cereals and cereal flour, bran and germ intended for direct human consumption (750, 75, 800, 3 µg kg^−1^), but also in cereal-based processing foods and baby foods for infants and young children (200, 20, 200, and 0.5 µg kg^−1^) in the European Union [23]. Moreover, the tolerable daily intakes (TDIs) for DON and its derivatives, ((15-acetyl-deoxynivalenol (15-AcDON), 3-acetyl-deoxynivalenol (3-AcDON), deoxynivalenol-3-glucoside (D_3_G)), nivalenol (NIV), ZEN, OTA, and FB1, are 1.0, 0.7, 0.25, 0.01, and 2 µg kg^−1^ bw day^−1^, respectively [24,25,26,27]. The European Food Safety Authority (EFSA) also released the thresholds of toxicological concern (TTC) for the genotoxic compounds AOH at 0.0025 µg kg^−1^ bw day^−1^ and for the non-genotoxic tentoxin (TEN) and TEA at 1500 µg kg^−1^ bw day^−1^ [28]. The probabilistic dietary risk exposure exceeded the safe chronic exposure levels at the 95P of DON through the intake of foods made from contaminated-wheat flours for teenagers in Brazil, which indicate differences in eating habits and body functions that present various dietary risks [29].

Wheat flours remain the predominant dietary source of mycotoxin exposure in grain consumption patterns [30]. However, studies on the co-occurrence of Alternaria and Fusarium toxins in wheat flours, their contamination differences between different types of wheat flour, and the exposure and toxicity of the Alternaria toxin are still limited. Targeted and continuous surveillance of contamination is necessary to explore contamination patterns, ensure minimal contamination and early prediction of their dietary exposure risks and their possible cumulative risks. The first objective of the present study was to survey and analyze the occurrence and co-occurrence of mycotoxins in wheat flours used for human consumption from Shanghai, China, 2020–2021, through a modified and validated UPLC-MS/MS method for the simultaneous determination of multiple mycotoxins (DON, 15-AcDON, 3-AcDON, D_3_G, NIV, NEO, FUS-X, AOH, TEA, TEN, OTA, ZEN, and FB_1_). The investigated data were used to compare distribution characteristics of mycotoxins in different types of wheat flours and to assess the chronic dietary intake risks for Shanghai residents in terms of age and gender. In particular, the approximate cumulative exposure risks of co-occurred mycotoxins in the same sample (>5%) were also considered for the first time.

## 2. Results and Discussion

### 2.1. Method Modification and Validation

The co-occurrence of Alternaria and Fusarium toxins in wheat flours has rarely been explored, and the method for simultaneous determination has been limited until now. After optimization, 75% acetonitrile with 1% formic acid and ACQUITY UPLC BEH C_18_ VanGuard pre-column with ACQUITY UPLC BEH C_18_ column were finally selected as the extraction agent and chromatography column with the acceptable extraction recoveries for most mycotoxins in wheat flour (60~120%) (Figure S1). Analytical parameters in the modified UPLC-MS/MS method were analyzed and validated. Values for the limit of detection (LOD) and limit of quantification (LOQ) for 13 mycotoxins were in the range of 0.08~62.5 µg kg^−1^ and 0.18~125 µg kg^−1^, which are far below the available MLs in foodstuffs. Acceptable linearities (R^2^ > 0.9) within the tested range were obtained (Table 1). Mycotoxin recovery in this method of fortified wheat flour samples at three levels ranged from 67.7% to 120.0% and the intra- and inter-day precisions were less than 20% (1.2~20.0%). The results of these validation parameters indicated that the modified method could be applied in the quantitative detection of mycotoxins in wheat flour samples.

### 2.2. Mycotoxins Occurrence in Wheat Flour Samples

#### 2.2.1. Mycotoxin Presence in Wheat Flour Samples

**Figure 1:**
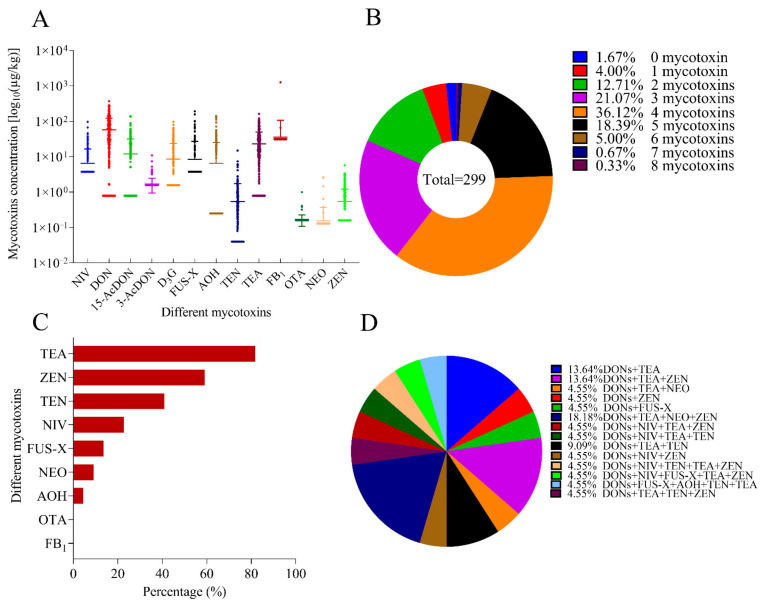
Mycotoxins occurrence in wheat flour samples. (**A**) occurrence of mycotoxins in wheat flour samples; (**B**) analytical results of mycotoxin coexistence in 299 wheat flour samples; (**C**) co-occurrence of mycotoxins in wheat flour samples (≥200 µg kg^−1^) contaminated with DONs; and (**D**) different combinations of the co-occurrence of mycotoxins in positive wheat flours for DONs (*n* = 22).

Detailed data on mycotoxin occurrence are shown in Table 2 and Figure 1A. The contamination levels for mycotoxins in wheat flour samples were lower than the existing regulations in cereals (95.6~100.0%), except for one sample. Among these mycotoxins, DON (74.9%), TEA (73.2%), TEN (55.2%), and ZEN (40.1%) had a higher detection rate in the wheat flours analyzed. The average contamination levels of DON, TEA, and FB_1_ were higher. The contamination levels of DON (4.4%) or OTA (0.3%) in some samples exceeded the limits of infant flour-based food. The detection rate and contamination level for OTA in this study were also lower than those in other results [18,19]. Only one of the 299 samples (1260.0 µg kg^−1^) exceeded the maximum limit of FB_1_ in maize and corn-based foods for direct human consumption, which contains 20% corn flour after further detailed examination. Exogenous food ingredients, such as corn or buckwheat, have possible effects on mycotoxin contamination in wheat flour.

The co-contamination rate of mycotoxins in wheat flours was as high as 98.1%, and more than half of the samples (57.2%) contained three to four mycotoxins (Figure 1B). The detailed combination of the co-occurrence of mycotoxins in wheat flours are shown in Table S1. There were four main contamination patterns: DON+15-AcDON+TEA+TEN (12.0%), DON + TEA + TEN (8.4%), NIV+DON+D_3_G+TEA+TEN (5.0%), and AOH+ZEN (5.0%). Among these samples with a total DON greater than 200, the detection rate of TEA, ZEN, TEN, NIV, FUS-X, NEO, or AOH in wheat flours was 81.8%, 59.1%, 40.9%, 22.7%, 13.6%, 9.1%, or 4.6%, respectively (Figure 1C,D). The co-contamination of Fusarium and Alternaria mycotoxin was relatively common, especially for the co-contamination of DON and TEA, which was also reported as the predominant contaminant pattern in the previous study [22].

#### 2.2.2. Distribution Characteristics and Differences of Mycotoxins in Wheat Flour Samples

**Figure 2:**
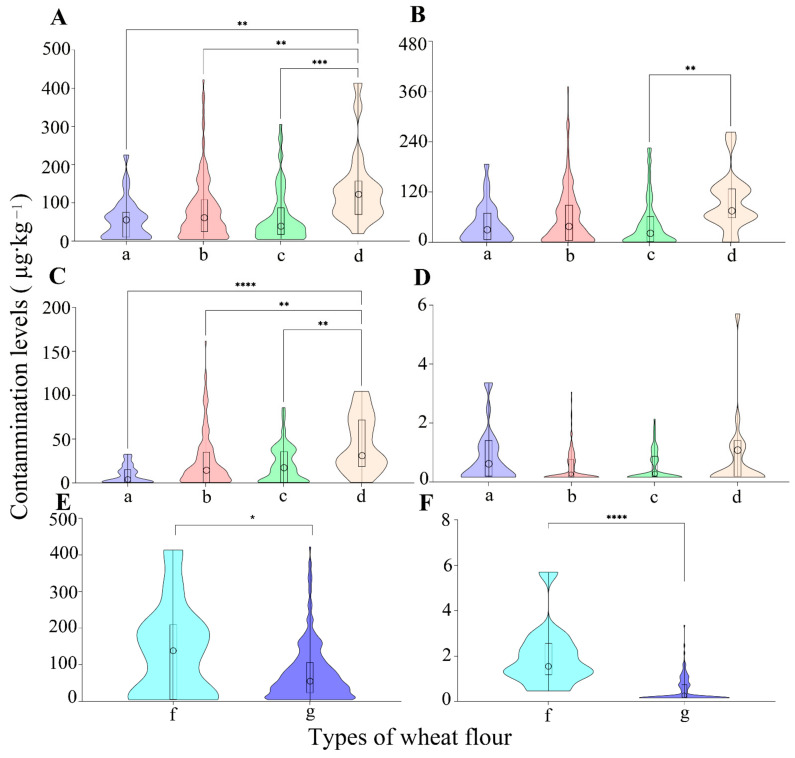
The distribution characteristics and differences of contamination levels for mycotoxins in various types of wheat flour samples (* *p* < 0.05, ** *p* < 0.01, *** *p* < 0.001 and **** *p* < 0.0001): (**A**) Occurrence of DONs in different wheat flour samples; (a) low-gluten wheat flours, (b) medium-gluten wheat flours, (c) high-gluten wheat flours, and (d) whole wheat flours; (**B**) occurrence of DON in different wheat flour samples; (a) low-gluten wheat flours, (b) medium-gluten wheat flours, (c) high-gluten wheat flours, and (d) whole wheat flours; (**C**) occurrence of TEA in different wheat flour samples; (a) low-gluten wheat flours, (b) medium-gluten wheat flours, (c) high-gluten wheat flours, and (d) whole wheat flours; (**D**) occurrence of ZEN in different wheat flour samples; (a) low-gluten wheat flours, (b) medium-gluten wheat flours, (c) high-gluten wheat flours, and (d) whole wheat flours; (**E**) occurrence of DONs in different wheat flour samples; (f) organic wheat flours, and (g) conventional wheat flours; and (**F**) occurrence of ZEN in different wheat flour samples; (f) organic wheat flours, and (g) conventional wheat flours.

We have carried out a comparative analysis for mycotoxin contamination in wheat flour involving wheat flour refining processing technology and organic agriculture production, especially from the perspective of whole vs. refined, low-gluten vs. medium-gluten vs. high-gluten, and organic vs. conventional wheat flours. The results showed that the average levels of tested mycotoxins in different types of wheat flours with different significance, except for NIV, FB_1_, NEO, and OTA (Figure 2 and Figure S2).

The total amount of DON in refined wheat flours with different protein content (56.1~80.3 µg kg^−1^) were significantly lower than that in whole wheat flour (138.4 µg kg^−1^), and the average level of DON in whole wheat flours (96.5 µg kg^−1^) was also higher than that in high-gluten wheat flours (43.4 µg kg^−1^) (Figure 2A,B). The similar contamination difference was also found for TEA, ZEN, FUS-X, or TEN between whole wheat flour and refined wheat flour (Figure 2C,D and Figure S2A,B). This distribution characteristic of DON or ZEN in whole wheat flours and refined wheat flours had also been found in a previous study, but the levels detected in this study were lower, which may be due to good management control [1]. Mycotoxin contamination levels in wheat flour were related to the proportions of different milling fractions because the epidermis, bran or germ of wheat grain as the natural medium of toxigenic fungi may concentrate mycotoxins. In other words, mycotoxins in wheat flours could be diminished by removing or adjusting grinding fractions with a high risk of contamination. Compared to low-gluten wheat flour, medium-gluten and high-gluten wheat flour had a higher degree and a wider range of contamination (Figure 2 and Figure S2). The similar patterns of contamination have been found in wheat flour related foods. Oueslati et al. reported that the contamination levels of combination (DON + ENB) in the Tunisian whole bread samples were higher than that in white bread samples [31]. The mycotoxin contamination (DON, TEA, and TEN) in noodles derived from high- or medium-gluten wheat flours were also found to be more serious than that in biscuits derived from low-gluten wheat flours [22]. In short, the monitoring of primary processed wheat flour should be regarded with more concern, even if contamination levels are below the limits, minimizing health risk as early as possible, especially for whole wheat flours, medium-gluten, and high-gluten wheat flours.

Meanwhile, there is still a controversy that organic wheat flour, without use of fertilizers and fungicides, contain more mycotoxins than conventional products, thereby involving another risk for human health because of limited studies on the comparation of organic and conventional wheat flours [8]. In this study, significant differences were only found in some mycotoxins and the average level of DON, ZEN, or AOH in organic wheat flours (135.9, 2.0, or 13.9 µg kg^−1^) was significantly higher than that in conventional wheat flours (77.3, 0.5, or 6.2 µg kg^−1^), while the mean contamination for TEN in organic wheat flours (0.05 µg kg^−1^) was exactly the opposite in conventional wheat flours (0.6 µg kg^−1^) (Figure 2E,F). DON, NIV, and FUS-X were reported to occur frequently in organic cereals from Italy [32]. Crop rotation, good agricultural, or harvest practices in terms of the proper transport and storage conditions was associated with mycotoxins contamination of organic wheat flours [9]. Therefore, mycotoxin of organic wheat flours was not always higher than that of conventional wheat flours, which needs further research considering the relationship of fungi and mycotoxin for variable environmental conditions.

### 2.3. Risk Assessment and Uncertainty of Ingestion or Exposure

**Figure 3:**
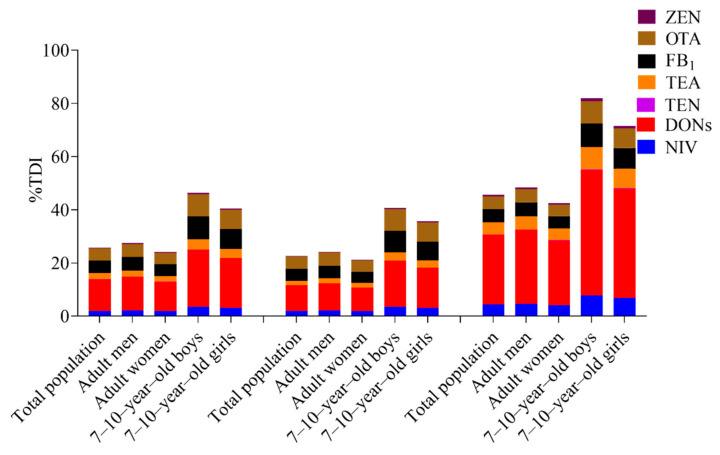
The health risk of various mycotoxins through wheat flour consumption were described in the upper bound deterministic and probabilistic estimation (Median and P90) for different consumer groups from left to right.

The deterministic and probabilistic assessment of exposure to each group of mycotoxins through wheat flour consumption for the local population were obtained (Table 3) by Equations (2) and (3) and are found in Section 4.4. The PDI values from the estimation of almost all mycotoxins were lower than the available *HBGVn*. In particular, the PDI values of AOH in the entire population ranged from 9.00 × 10^−3^ to 7.88 × 10^−2^ µg kg^−1^ bw day^−1^, which were higher than the recommended TTC values for AOH at 0.0025 µg kg^−1^ bw day^−1^. The %TDI ranking results of assessments were almost the same as AOH > DONs > FB_1_ > OTA > TEA > NIV > ZEN > TEN. The %TDI or sum of %TDI from the single exposure assessment of almost all mycotoxins, except for AOH, were less than 100% in all populations (Table 3 and Figure 3), indicating that wheat flour consumption for Shanghai residents contributed little to the exposure risk of mycotoxins. The margin of exposure values were the ratios of BMDL_10_ values of 4.7 and 14.5 µg kg^−1^ bw day^−1^ to *PDI*, respectively, reflecting the nonneoplastic and neoplastic effects of OTA. The results showed that they both exceeded 200 and 10,000, indicating no health problems (Table S2).

Considering mycotoxins co-contamination, the approximate cumulative exposure risks were also assessed in positive samples contaminated with multiple mycotoxins concurrently and their incidence greater than 5%. Among these four main contamination patterns, only the %TDI min or %TDI max from one of patterns (AOH + ZEN) was more than 100% (Table S3). It is important to note that limited knowledge is available on the transfer or fate of mycotoxins during food processing and digestion. It is reported that 60~80% of OTA and AFs in wheat flour can be retained after cooking [33]. The fates of mycotoxins, particularly DON and D_3_G, in bread making are affected by fermentation or other complex factors [2,6,34]. Some externally additive food compositions can also affect the risk of eventual exposure to mycotoxins. Recently, bread enriched with pumpkin extract and fermented whey individually and in combination were reported to reduce the bio-accessibilities of mycotoxins and alleviate their associated neurotoxicity [35,36,37]. Therefore, the associated health risks of AOH needs to be further studied, in combination with internal exposure, especially in children. A recent study also pointed to the need to pay attention to the risks of infant exposure to AOH through cereal-based foods consumption [22].

## 3. Conclusions

Wheat flour is one of the important dietary sources of mycotoxin exposure and its safety deserves attention. We inspected 13 mycotoxins in wheat flours marketed in Shanghai by UPLC-MS/MS and profiled their external exposure risk. Of the wheat flour samples with low concentration levels, 95.6~100.0% met the regulations. The average contamination levels of DON and TEA in wheat flours were higher than other mycotoxins. Of the wheat flour samples, 57.2% were contaminated simultaneously by three to four mycotoxins. We found the co-occurrence of Fusarium and Alternaria mycotoxin in wheat flours. Particularly, four main co-contamination patterns in wheat flours were also found in this study, including DON+15-AcDON+TEA+TEN, DON+TEA+TEN, NIV+DON+D_3_G+TEA+TEN, and AOH+ZEN. Lower average contamination levels and fewer types of mycotoxins were detected in refined flours than whole wheat flours. Combined with the relevant wheat food consumption data of Shanghai residents, chronic dietary intake risk assessments of mycotoxins were performed by point evaluation and Monte Carlo assessment model. Dietary exposure risks of DONs, ZEN, NIV, TEA, TEN, FB_1_, and OTA by wheat flours intakes were considered to be acceptable (%TDI < 100%). However, the exposure risk of AOH and the approximate cumulative exposure risks of AOH and ZEN should be considered and further studied in conjunction with internal exposure assessments.

## 4. Materials and Methods

### 4.1. Materials and Reagents

The ZEN (Z2125), DON (D0156), 3-AcDON (A6166), 15-AcDON (A1556), D_3_G (32911), OTA (O1877), AOH (A1312), TEN (35977), NIV (34131), and FB_1_ (F1147) analytical standards are Sigma-Aldrich products (St. Louis, MO, USA). The TEA (ab142764) analytical standards were purchased from Abcam (Cambridge, MA, USA). The FUS-X (10003647) and NEO (10003640) analytical standards are Romer Lab Biopure™ products (Union, MO, USA). The certified wheat flour (Fusarium mycotoxins, ERMBC600) as the reference material for quality control was also obtained from Sigma-Aldrich (St. Louis, MO, USA). HPLC-grade acetonitrile was purchased from Merck (Darmstadt, Germany). Milli-Q quality water (Millipore, Billerica, MA, USA) was used throughout the experiments.

### 4.2. Sampling and Samples for Analysis

Two hundred and ninety-nine wheat flour samples were randomly taken from retail stores or supermarkets located in different zones of Shanghai from December 2020 to October 2021. These samples originated from twelve different districts representing central and suburban Shanghai, including Congming (*n* = 21), Changning (*n* = 38), Huangpu (*n* = 29), Hongkou (*n* = 8), Jingan (*n* = 25), Minhang (*n* = 47), Pudong (*n* = 29), Putuo (*n* = 30), Qingpu (*n* = 10), Songjiang (*n* = 10), Xuhui (*n* = 46), and Yangpu (*n* = 6). Of the samples, 13 out of 299 were bulk wheat flours, while the remaining wheat flour samples were categorized as whole wheat flours and refined wheat flours according to labels. According to the labeled varying content of the protein, refined wheat flours involved low-gluten wheat flours (*n* = 29), medium-gluten wheat flours (*n* = 166), and high-gluten wheat flours (*n* = 71). Almost all of them were packaged in plastic food containers at least 500 g/sample. 13 of 299 samples were organic wheat flours after a subdivision of the collected samples in terms of agricultural practice. The geographic origin, food composition, and other information about these samples used for humans were also recorded. All the collected samples were divided and stored in plastic cans at −20 °C before analysis.

### 4.3. Analytical Method and Validation

A total of 13 mycotoxins, including DON, 3-AcDON, 15-AcDON, D_3_G, NIV, FUS-X, NEO, ZEN, OTA, FB_1_, AOH, TEA, and TEN, were simultaneous determined in wheat flours following the UPLC-MS/MS method reported by previous studies [22,38] with some modifications. First, more Fusarium toxins and Alternaria toxins were monitored in the full-scan mode. The extraction recoveries for targeted mycotoxins were compared under different concentrations of organic reagents after adding 13 mycotoxins simultaneously in wheat flour samples at middle concentration levels. The equation for calculating their recoveries, where *Ca* is the calculated concentration in the mycotoxins-spiked sample, *Cb* is the calculated concentration in the non-spiked sample, and CA is the theoretical concentration of the analyte that was added into the sample.

Recovery (%) = (*Ca* − *Cb*)/CA × 100 (1)

In brief, 1.0 g of sample was vortexed vigorously for 5 min using 4 mL acetonitrile: water (75:25, *v*/*v*) with 1% formic acid solution, and then ultrasound-assisted extracted for 40 min. The extraction mixture was centrifuged at 4000 rpm for 5 min. After centrifugation, 2 mL of the supernatant extract was filtered with 0.22 μm organic filter membrane and injected into the ultimate 3000 UPLC system (Thermo Fisher Scientific, San Jose, CA, USA). Mycotoxins were separated by the ACQUITY UPLC BEH C_18_ VanGuard pre-column (1.7 µm, 2.1 mm × 5 mm) and ACQUITY UPLC BEH C_18_ (1.7 µm, 2.1 mm × 100 mm) column with mobile phase A (water containing 5 mM ammonium acetate) and mobile phase B (methanol) gradient elution following: 0–1.0 min, 5–50% B; 1.0–9.0 min, 50–100% B; 9.0–10.0 min, 100% B; 10.0–11.0 min, 100–5% B; and 11.0–12.0 min, 5% B. The flow rate was 0.35 mL min^−1^ and the injection volume was 5 μL. Meanwhile, the TSQ VantageTM (Thermo Fisher Scientific, San Jose, CA, USA) triple stage quadrupole mass spectrometer was applied for further multi-mycotoxin determination based on multiple reaction monitoring (MRM) using positive and negative electrospray ionization (ESI^+/−^) mode. Based on the selected optimal parent ions, the product ion and their optimized collision energies with argon for each mycotoxin were obtained and summarized in the Table S4. Other optimized parameters were set as follows: Positive spray voltage at +3.0 kV, negative spray voltage at −2.5 kV, capillary temperature at 300 °C, vaporizer temperature at 250 °C, aux gas pressure is 5 psi, and sheath gas pressure is 40 psi. Their final chromatograms is shown in the Figure S3. Related parameters of the UPLC-MS/MS method were verified with the guidelines of the document [39]. Linearity was determined by analyzing each mycotoxin standard solutions. The values (LOD and LOQ) for mycotoxins were determined by the signal-to-noise (S/N) ratios of 3:1 and 10:1, respectively, according to the lowest detectable level for quantitative ion [1,35]. Recovery analysis was conducted using three different concentrations of 13 mycotoxins to fortify simultaneously wheat flour matrices. The spiked concentration ranged from 0.13 to 1200 µg kg^−1^ with six replicates per concentration level (Table 1). The precision and accuracy of the proposed strategy were checked through intra- and inter-day analysis, as described in our previous study, and RSD r or RSD_R_ less than 20% was evaluated as acceptable [39,40].

### 4.4. Dietary Risk Assessment and Characterization

The risk of exposure for ingested mycotoxins through wheat flour consumption was assessed by the deterministic and probabilistic approach. The probable daily intake (*PDI n*, µg kg^−1^ bw day^−1^) of each mycotoxin was calculated by their contamination levels obtained from the analyzed samples combined with the relevant wheat food consumption data, as indicated in the following equation:

*PDI n* = (*Cn* × CA)/BW (2)

For the point evaluation, where *Cn* is the average content of each mycotoxin *n*. In this study, if a contamination value was not detected, which refers to values lower than LOD values, the substitution values of 0 (lower bound), 1/2 LOD (middle bound) and LOD (upper bound) were used for mycotoxin exposure assessment. CA is the average consumption amount of the commodity (g person^−1^ day^−1^) and BW is the average body weight of participants (kg). Wheat and wheat-based products consumption data and the demographic information were derived from a 2012~2014 Shanghai Food Consumption Survey (SHFCS) by Fudan University regarding to Shanghai inhabitants (7~60 years old). The average consumption of participants in 7~10 years old groups was the highest in the published study [13]. Therefore, the population groups considered in this study were consistent with a previous study [16]: Total population, adult men, adult women, as well as typical boys and girls (Table S5).

A more accurate or applicable Monte Carlo simulation was also performed using @Risk Industrial 7.5 (Palisade, New York, NY, USA) software for probability assessment, in combination with Microsoft Excel 2016. Mycotoxin contamination data of all analyzed samples at the above three bounds were input into @RISK software and *Cn* could be obtained from the best-fitting distribution for these data. The Anderson-Darling and Kolmogorov-Smirnov tests were selected to evaluate the goodness-of-fit for each distribution by @Risk software. Similarly, *Cn*, CA, and BW were input into @RISK software according to the above formula for Monte Carlo simulation. The exposure distribution of *PDI n* with a confidence interval > 90% were obtained using 10,000 iteration runs. The health risk characterization of each mycotoxin (%*TDI n*) was performed by dividing *PDI n* with their health-based guidance values (*HBGV n*) based on the equation shown below:

%*TDI n* = (*PDI n*/*HBGV n*) × 100 (3)

where *HBGV n* represents the available *TDI n* or *TTC n*. Values of %*TDI n* higher than a hundred indicate a possible health risk scenario. Otherwise, there is no significant risk was observed and a population is not at risk from that exposure. An approximation of exposure assessment was also performed to evaluate consumer’s exposure in analyzed samples contaminated with multiple mycotoxins [41]. The *Cn*, *min* and *Cn*, *max* derived from multi-mycotoxin contaminated samples. Then, we summed them, and a combined health risk characterization was proposed as follows:

(4) $$\overset{i}{\underset{m=1}{\sum }}\%TDI\;n,min=\overset{i}{\underset{m=1}{\sum }}\left(Cn,min\;\times \;CA\right)/Bw/HBGVn$$

(5) $$\overset{i}{\underset{m=1}{\sum }}\%TDI\;n,max=\overset{i}{\underset{m=1}{\sum }}\left(Cn,max\;\times \;CA\right)/Bw/HBGVn$$

Conventionally, values of $\overset{i}{\underset{m=1}{\sum }}\%TDI$ less than a hundred indicate that the combined exposure level were considered to be acceptable, and people are unlikely to be exposed at a toxic level with possible consequences for health.

### 4.5. Data Analysis

The UPLC-MS raw data were recognized by Thermo Xcalibur Qual Browser 4.0. Nonparametric statistics were used after the normality and lognormality testing for each group. Distribution characteristics and differences of mycotoxins in various types of wheat flour samples were compared and evaluated using the Kruskal-Wallis or Mann-Whitney test at a significance level of 0.05. All statistical analyses and drawings were performed using GraphPad Prism 9.0 software.

## Figures and Tables

**Table 1:** Overview of the accuracy and precision of the developed LC-MS/MS method.

Mycotoxin	Spike (µg kg^−1^)	Recovery ± RSD r (%, *n =* 6)	Recovery ± RSD_R_ (%, *n =* 6)	Linearity Equation (R^2^)	LOD (µg kg^−1^)	LOQ (µg kg^−1^)
NIV	50	76.4 ± 19.8	85.7 ± 17.5	Y = 1.9X (0.9891)	7.5	25
	100	106.4 ± 19.8	96.2 ± 18.5			
	200	71.5 ± 17.8	110.9 ± 15.2			
DON	50	115.0 ± 4.5	73.6 ± 7.4	Y = 17.1X (0.9258)	1.6	3.1
	100	109.4 ± 7.1	67.7 ± 5.1			
	200	105.4 ± 5.9	100.0 ± 6.5			
15-AcDON	50	96.1 ± 15.4	119.9 ± 3.6	Y = 36.8X (0.9971)	1.6	3.1
	100	109.6 ± 10.1	118.0 ± 5.5			
	200	91.5 ± 9.5	120.0 ± 1.2			
3-AcDON	50	111.9 ± 4.6	114.5 ± 5.9	Y = 27.0X (0.9949)	3.1	6.2
	100	111.2 ± 6.1	114.6 ± 2.7			
	200	116.6 ± 3.3	118.6 ± 3.3			
D_3_G	50	85.4 ± 19.3	119.0 ± 6.6	Y = 9.7X (0.9956)	3.1	6.2
	100	98.7 ± 8.1	117.1 ± 5.9			
	200	81.7 ± 12.7	108.9 ± 14.3			
FUS-X	50	105.7 ± 6.3	102.9 ± 9.5	Y = 43.1X (0.9972)	7.5	25
	100	109.5 ± 7.2	110.7 ± 5.3			
	200	80.7 ± 4.4	118.3 ± 5.3			
OTA	10	92.2 ± 5.7	113.6 ± 2.6	Y = 1271.2X (0.9889)	0.3	0.6
	20	108.7 ± 7.0	102.0 ± 12.6			
	40	83.8 ± 6.3	115.9 ± 9.8			
NEO	1	102.0 ± 14.8	119.5 ± 10.4	Y = 529.7X (0.9850)	0.2	0.5
	2	84.9 ± 18.7	115.8 ± 6.7			
	4	83.1 ± 16.8	118.7 ± 6.7			
ZEN	10	92.3 ± 6.5	85.6 ± 6.7	Y = 530.9X (0.9976)	0.3	0.6
	20	112.4 ± 5.5	117.4 ± 5.6			
	40	98.4 ± 3.3	104.5 ± 9.2			
AOH	30	82.3 ± 20.0	106.8 ± 9.9	Y = 38.4X (0.9964)	0.5	1.0
	60	91.8 ± 15.4	115.2 ± 11.4			
	120	74.6 ± 11.8	118.0 ± 5.1			
TEA	50	95.4 ± 12.8	113.2 ± 7.8	Y = 52.7X (0.9934)	1.6	3.1
	100	91.1 ± 8.7	108.3 ± 10.1			
	200	79.3 ± 10.0	104.3 ± 4.7			
TEN	5	99.6 ± 5.8	111.2 ± 6.4	Y = 1875.8X (0.9844)	0.08	0.2
	10	105.4 ± 4.9	91.2 ± 13.8			
	20	100.8 ± 4.2	106.2 ± 10.5			
FB_1_	250	109.1 ± 7.4	98.4 ± 14.1	Y = 5.4X (0.9949)	62.5	125
	500	107.0 ± 7.9	87.5 ± 19.5			
	1000	110.1 ± 5.0	105.4 ± 7.2			

Low, Middle, and High represent the spiked low, middle, and high concentrations of mycotoxins respectively. RSD r: intraday precision (repeatability) in mycotoxin-fortified samples; RSD_R_: inter-day precision (reproducibility) in mycotoxin-fortified samples.

**Table 2:** Concentration levels of mycotoxins in wheat flour samples from Shanghai, China, 2020–2021 (*n* = 299, µg kg^−1^).

Mycotoxins	MRLs	Positive Rate (%)	Below MRLs (%)	Mean ± SD	Range
DON	750 ^a^, 200 ^b^	74.9	100.0, 95.6	57.6 ± 65.3	0.8–371.4
15-Ac DON	NF	37.8	**	12.0 ± 19.8	0.8–140.6
3-Ac DON	NF	4.0	**	1.7 ± 0.8	1.6–10.8
D_3_G	NF	32.1	**	8.6 ± 15.0	1.6–96.3
FUS-X	NF	11.7	**	8.5 ± 18.8	3.6–191.7
NIV	NF	13.7	**	6.6 ± 9.8	3.8–96.7
AOH	NF	18.4	**	6.6 ± 18.7	0.2–140.8
TEN	NF	55.2	**	0.5 ± 1.2	0.04–14.8
TEA	NF	73.2	**	23.1 ± 27.0	0.8–161.6
ZEN	75 ^a^, 20 ^b^	40.1	100.0, 100.0	0.6 ± 0.7	0.2–5.7
FB_1_	800 ^a^, 200 ^b^	0.7	99.7, 99.7	35.5 ± 71.1	31.2–1260.4
OTA	3 ^a^, 0.5 ^b^	2.7	100.0, 99.7	0.2 ± 0.06	0.2–1.0
NEO	NF	2.3	**	0.6 ± 0.2	0.1–2.6

MRLs, Maximum Regulation Limits; SD, Standard Deviation; NF, Not Found; **, No Data. ^a^ MRLs for DON, ZEN, FB1, and OTA in cereal flour, maize-based breakfast cereals, or processed cereals as end product marketed for direct human consumption (EC Regulation No 1881/2006). ^b^ MRLs for DON, ZEN, FB1, and OTA in processed cereal-based foods and baby foods for infants and young children (EC Regulation No 1881/2006).

**Table 3:** Exposure levels of mycotoxins in wheat flour samples with the upper bound deterministic and probabilistic estimation for different consumer groups (µg kg^−1^ bw day^−1^).

	Mycotoxins	Deterministic Estimation	Probabilistic Estimation	
Poulation			Median	P90
NIV (TDI, 0.7 µg kg^−1^ bw day^−1^)				
Total population		1.40 × 10^−2^	1.40 × 10^−2^	3.04 × 10^−2^
Adult men		1.49 × 10^−2^	1.49 × 10^−2^	3.24 × 10^−2^
Adult women		1.31 × 10^−2^	1.31 × 10^−2^	2.84 × 10^−2^
7–10-year-old boys		2.52 × 10^−2^	2.52 × 10^−2^	5.47 × 10^−2^
7–10-year-old girls		2.20 × 10^−2^	2.20 × 10^−2^	4.78 × 10^−2^
DONs (TDI, 1 µg kg^−1^ bw day^−1^)				
Total population		1.18 × 10^−1^	9.58 × 10^−2^	2.62× 10^−1^
Adult men		1.26 × 10^−1^	1.02× 10^−1^	2.79× 10^−1^
Adult women		1.11 × 10^−1^	8.94 × 10^−2^	2.45× 10^−1^
7–10-year-old boys		2.13 × 10^−1^	1.72× 10^−1^	4.71× 10^−1^
7–10-year-old girls		1.86 × 10^−1^	1.50× 10^−1^	4.12× 10^−1^
AOH (TTC, 0.0025 µg kg^−1^ bw day^−1^)				
Total population		9.64 × 10^−3^	9.64 × 10^−3^	4.38 × 10^−2^
Adult men		1.03 × 10^−2^	1.03 × 10^−2^	4.66 × 10^−2^
Adult women		9.00 × 10^−3^	9.00 × 10^−3^	4.09 × 10^−2^
7–10-year-old boys		1.73 × 10^−2^	1.73 × 10^−2^	7.88 × 10^−2^
7–10-year-old girls		1.51 × 10^−2^	1.51 × 10^−2^	6.88 × 10^−2^
TEN (TTC, 1.5 µg kg^−1^ bw day^−1^)				
Total population		7.69 × 10^−4^	5.37 × 10^−4^	1.65 × 10^−3^
Adult men		8.18 × 10^−4^	5.72 × 10^−4^	1.75 × 10^−3^
Adult women		7.18 × 10^−4^	5.01 × 10^−4^	1.54 × 10^−3^
7–10-year-old boys		1.38 × 10^−3^	9.66 × 10^−4^	2.96 × 10^−3^
7–10-year-old girls		1.21 × 10^−3^	8.44 × 10^−4^	2.59 × 10^−3^
TEA (TTC, 1.5 µg kg^−1^ bw day^−1^)				
Total population		3.31 × 10^−2^	2.60 × 10^−2^	7.00 × 10^−2^
Adult men		3.52 × 10^−2^	2.76 × 10^−2^	7.45 × 10^−2^
Adult women		3.08 × 10^−2^	2.42 × 10^−2^	6.53 × 10^−2^
7–10-year-old boys		5.94 × 10^−2^	4.67 × 10^−2^	1.26 × 10^−1^
7–10-year-old girls		5.19 × 10^−2^	4.08 × 10^−2^	1.10 × 10^−1^
FB_1_ (TDI, 2 µg kg^−1^ bw day^−1^)				
Total population		9.53 × 10^−2^	8.95 × 10^−2^	9.88 × 10^−2^
Adult men		1.01× 10^−1^	9.52 × 10^−2^	1.05 × 10^−1^
Adult women		8.89 × 10^−2^	8.35 × 10^−2^	9.21 × 10^−2^
7–10-year-old boys		1.71 × 10^−1^	1.61 × 10^−1^	1.78 × 10^−1^
7–10-year-old girls		1.50 × 10^−1^	1.41 × 10^−1^	1.55 × 10^−1^
OTA (TDI, 0.01 µg kg^−1^ bw day^−1^)				
Total population		4.64 × 10^−4^	4.58 × 10^−4^	4.67 × 10^−4^
Adult men		4.93 × 10^−4^	4.88 × 10^−4^	4.97 × 10^−4^
Total population		4.33 × 10^−4^	4.28 × 10^−4^	4.36 × 10^−4^
7–10-year-old boys		8.34 × 10^−4^	8.24 × 10^−4^	8.40 × 10^−4^
7–10-year-old girls		7.28 × 10^−4^	7.20 × 10^−4^	7.33 × 10^−4^
ZEN (TDI, 0.25 µg kg^−1^ bw day^−1^)				
Total population		7.88 × 10^−4^	6.17 × 10^−4^	1.59 × 10^−3^
Adult men		8.38 × 10^−4^	6.57 × 10^−4^	1.69 × 10^−3^
Adult women		7.35 × 10^−4^	5.76 × 10^−4^	1.48 × 10^−3^
7–10-year-old boys		1.42 × 10^−3^	1.11 × 10^−3^	2.85 × 10^−3^
7–10-year-old girls		1.24 × 10^−3^	9.70 × 10^−4^	2.49 × 10^−3^

## Data Availability

The data that support the findings of this study are available from the corresponding author upon reasonable request.

## Supplementary Materials

The following supporting information can be downloaded at: https://www.mdpi.com/article/10.3390/toxins14110748/s1, Figure S1. The effect of the extractant types containing 1% formic acid on the recoveries of mycotoxins: (a) 45% Acetonitrile, (b) 55% Acetonitrile, (c) 65% Acetonitrile, (d) 75% Acetonitrile, (e) 85% Acetonitrile, (f) 95% Acetonitrile. The two dashed lines represent recoveries of 60% and 120%, respectively; Figure S2. The distribution characteristics and differences of contamination levels for mycotoxins in various types of wheat flour samples: (A) occurrence of FUS-X in different wheat flour samples; (a) low-gluten wheat flours, (b) medium-gluten wheat flours, (c) high-gluten wheat flours, (d) whole wheat flours; (B) occurrence of TEN in different wheat flour samples; (a) low-gluten wheat flours, (b) medium-gluten wheat flours, (c) high-gluten wheat flours, (d) whole wheat flours; (C) occurrence of AOH in different wheat flour samples; (f) organic wheat flours, g) conventional wheat flours; (D) occurrence of TEN in different wheat flour samples; (f) organic wheat flours, (g) conventional wheat flours; Figure S3. The chromatograms of 13 mycotoxins at middle concentration under optimized chromatographic and mass spectrometry conditions; Table S1. Different combinations of co-occurrence of mycotoxins in wheat flours marketed in China; Table S2. The nonneoplastic and neoplastic effects through wheat flours consumption based on various margin of exposure estimation values of OTA; Table S3. The approximate cumulative exposure risks of four main contamination patterns of mycotoxins co-occurring in real samples for the total population; Table S4. Retention time and MS parameters for the analysis of mycotoxins; Table S5. The average daily intake of wheat and wheat-based products in Shanghai and participants for average body weight.
