# Supplementary material for: Mycotoxins in Wheat Flours Marketed in Shanghai, China: Occurrence and Dietary Risk Assessment
Source: Toxins (Basel). 2022 Oct 31;14(11):748. doi: 10.3390/toxins14110748 (PMC9698038; doi:10.3390/toxins14110748)
Supplement: Supplementary file 1 [file toxins-14-00748-s001.zip › toxins-1942709-supplementary.pdf]

toxins

MDPI

Supplementary Materials: Mycotoxins in Wheat Flours Marketed in Shanghai, China: Occurrence and Dietary Risk Assessment

Haiyan Zhou, Anqi Xu, Meichen Liu, Zheng Yan, Luxin Qin, Hong Liu, Aibo Wu and Na Liu

Table S1. Different combinations of co-occurrence of mycotoxins in wheat flours marketed in China.

N	Incidence	Frequency (%)	N	Incidence	Frequency (%)
2			4		
D_3G+DON	2	0.67	NIV+AOH+ZEN+3-AcDON	1	0.33
DON+TEA	7	2.34	NIV+AOH+ZEN+15-AcDON	1	0.33
15-AcDON+TEA	2	0.67	DON+TEA+TEN+FUS-X	2	0.67
DON+15-AcDON	2	0.67	DON+FUS-X+AOH+ZEN	1	0.33
DON+FUS-X	1	0.33	15-AcDON+D_3G+AOH+ZEN	2	0.67
TEA+ZEA	2	0.67	FUS-X+D_3G+AOH+ZEN	1	0.33
AOH+ZEN	15	5.02	FUX+15-AcDON +AOH+ZEN	1	0.33
3			15-AcDON +AOH+ZEN+OTA	1	0.33
DON+15-AcDON +TEA	5	1.67	DON+AOH+TEN+TEA	1	0.33
DON+15-AcDON +FUS-X	1	0.33	DON+AOH+TEN+ZEN	1	0.33
DON+15-AcDON +TEN	1	0.33	5		
DON+TEA+FUS-X	1	0.33	DON+15-AcDON +D_3G+TEA+ZEN	5	1.67
DON+TEA+TEN	25	8.36	DON+15-AcDON +D_3G+TEA+NEO	1	0.33
DON+TEA+D_3G	1	0.33	DON+15-AcDON +D_3G+TEA+TEN	3	1.00
DON+TEA+OTA	1	0.33	DON+15-AcDON +D_3G+AOH+ZEN	1	0.33
DON+TEA+ZEN	3	1.00	DON+15-AcDON +FUS-X+TEA+TEN	5	1.67
DON+D_3G+ZEN	4	1.34	DON+15-AcDON +TEA+TEN+ZEN	2	0.67
DON+AOH+TEN	1	0.33	DON+15-AcDON +TEA+TEN+AOH	2	0.67
DON+FUS-X+ZEN	1	0.33	DON+15-AcDON +TEA+TEN+NEO	2	0.67
AOH+D_3G+ZEN	6	2.01	NIV+DON+15-AcDON +TEA+TEN	1	0.33
NIV+AOH+ZEN	2	0.67	DON+15-AcDON +3-AcDON+TEA+TEN	1	0.33
NIV+TEA+ZEN	1	0.33	DON+15-AcDON +3-AcDON+TEA+ZEN	1	0.33
15-AcDON +D_3G+TEA	1	0.33	DON+15-AcDON+TEA+FB_1+ZEN	1	0.33
15-AcDON +TEA+TEN	1	0.33	NIV+DON+D_3G+TEA+TEN	15	5.02
15-AcDON +TEA+ZEN	2	0.67	NIV+DON+TEA+TEN+FB_1	1	0.33
15-AcDON +AOH+ZEN	1	0.33	DON+D_3G+TEA+TEN+ZEN	7	2.34
AOH+TEA+ZEN	2	0.67	DON+D_3G+FUS-X+TEA+TEN	1	0.33
FUS-X+AOH+ZEN	1	0.33	DON+FUS-X+TEA+TEN+ZEN	1	0.33
D_3G+OTA+ZEN	1	0.33	DON+3Ac-DON+TEA+TEN+ZEN	1	0.33
AOH+TEN+ZEN	1	0.33	NIV+DON+FUS-X+AOH+ZEN	1	0.33
4			15-AcDON+TEN+TEA+OTA+NEO	1	0.33
DON+15-AcDON +D_3G+FUS-X	2	0.67	FUS-X+TEN+TEA+ZEN+OTA	1	0.33
DON+15-AcDON +D_3G+TEA	5	1.67	15-AcDON +FUS-X+AOH+OTA+ZEN	1	0.33
DON+15-AcDON +D_3G+ZEN	2	0.67	6		
DON+15-AcDON +D_3G+TEN	1	0.33	DON+15-AcDON +FUS-X+TEN+TEA+D_3G	1	0.33
DON+15-AcDON +TEA+TEN	36	12.04	DON+15-AcDON +FUS-X+TEN+TEA+NEO	1	0.33
DON+15-AcDON +TEA+ZEN	3	1.00	DON+15-AcDON +FUS-X+TEN+TEA+NIV	1	0.33

DON+15-AcDON +TEA+NEO	1	0.33	DON+15-AcDON +FUS-X+TEN+TEA+AOH	1	0.33
DON+15-AcDON +TEN+OTA	1	0.33	NIV+DON+D_3G+TEN+TEA+ZEN	2	0.67
NIV+DON+15-AcDON +TEN	1	0.33	DON+D_3G+3-AcDON+TEN+TEA+ZEN	1	0.33
DON+TEA+TEN+ZEN	8	2.68	DON+D_3G+15-AcDON +TEN+TEA+ZEN	1	0.33
DON+D_3G+TEA+TEN	12	4.01	DON+3-AcDON +FUS-X+TEN+TEA+ZEN	1	0.33
DON+D_3G+TEA+ZEN	5	1.67	NIV+DON+3-AcDON +TEN+TEA+ZEN	1	0.33
DON+D_3G+AOH+ZEN	2	0.67	DON+15-AcDON +D_3G+TEA+NEO+ZEN	1	0.33
NIV+DON+D_3G+ZEN	1	0.33	DON+15-AcDON +D_3G+FUS-X+TEA+ZEN	1	0.33
DON+D_3G+TEN+ZEN	1	0.33	DON+15-AcDON +AOH+TEN+TEA+ZEN	1	0.33
ZEN+AOH+TEA+FUS-X	1	0.33	NIV+DON+D_3G+FUS-X+TEA+ZEN	1	0.33
ZEN+AOH+TEA+D_3G	2	0.67	NIV+DON+3-AcDON +FUS-X+TEN+TEA	1	0.33
ZEN+AOH+TEA+DON	2	0.67	7		
ZEN+AOH+TEA+3-AcDON	1	0.33	NIV+DON+D_3G+FUS-X+TEN+TEA+ZEN	1	0.33
ZEN+TEA+DON+FUS-X	1	0.33	NIV+DON+15-AcDON +3-AcDON +D_3G+TEA+ZEN	1	0.33
ZEN+TEA+15-AcDON+D_3G	1	0.33	8		
NIV+DON+TEA+TEN	7	2.34	NIV+DON+15-AcDON +3-AcDON +D_3G+FUS-X+TEN+TEA	1	0.33

Table S2. The nonneoplastic and neoplastic effects through wheat flours consumption based on various margin of exposure estimation values of OTA.

Wheat Flours	*PDI* $\mu g \cdot kg^{-1}$ $bw \cdot day^{-1}$	nonneoplastic_BMDL10 / *PDI*	neoplastic_BMDL10 / *PDI*
The upper bound deterministic estimation			
Total population	4.64×10^{-4}	10,198.60	31,264.20
Adult men	4.93×10^{-4}	9587.59	29,391.14
Total population	4.33×10^{-4}	10,931.11	33,509.75
7–10-year-old boys	8.34×10^{-4}	5671.90	17,387.45
7–10-year-old girls	7.28×10^{-4}	6494.72	19,909.81
The upper bound probabilistic estimation (Median)			
Total population	4.58×10^{-4}	10,318.72	31,632.45
Adult men	4.88×10^{-4}	9700.57	29,737.49
Total population	4.28×10^{-4}	11,059.93	33,904.65
7–10-year-old boys	8.24×10^{-4}	5738.69	17,592.18
7–10-year-old girls	7.20×10^{-4}	6571.18	20,144.20
The upper bound probabilistic estimation (P90)			
Total population	4.67×10^{-4}	10,126.74	31,043.93
Adult men	4.97×10^{-4}	9520.17	29,184.45
Total population	4.36×10^{-4}	10,854.10	33,273.67
7–10-year-old boys	8.40×10^{-4}	5631.96	17,264.99
7–10-year-old girls	7.33×10^{-4}	6448.97	19,769.58

Table S3. The approximate cumulative exposure risks of four main pollution patterns of mycotoxins co-occurring in real samples for the total population.

Co-occurrence	C min	C max	%TDI min	%TDI max
DON+15-AcDON+TEA+TEN	15.10	317.94	2.07	43.06
DON+TEA+TEN	9.8	230.27	1.20	27.11
NIV+DON+D3G+TEA+TEN	86.81	370.82	12.41	48.83
AOH+ZEN	14.50	95.29	**796.23**	**5302.32**

Table S4. Retention time and MS parameters for the analysis of mycotoxins.

Mycotoxin	Molecular Weight	TR (min)	Molecular Ion	ESI	Parent Ions (m/z)	Product Ions (m/z)	CE (eV)
NIV	312.31	2.95	$[M+CH_3COO]^-$	ESI^-	371.00	281.00	18
					371.10	59.00	10
DON	296.32	3.30	$[M+CH_3COO]^-$	ESI^-	355.00	265.00	17
					355.10	247.20	22
3-AcDON	338.35	4.06	$[M+CH_3COO]^-$	ESI^-	397.00	307.16	16
					397.10	173.10	15
15-AcDON	338.35	4.10	$[M+NH_4]^+$	ESI^+	356.10	321.00	13
					356.00	137.00	5
D_3G	458.46	3.20	$[M+CH_3COO]^-$	ESI^-	517.10	457.40	14
					517.00	427.40	22
FUS-X	354.36	3.52	$[M+H]^+$	ESI^+	355.04	175.00	18
					355.03	137.00	17
OTA	403.81	5.46	$[M+H]^+$	ESI^+	404.12	221.00	35
					404.11	105.10	18
NEO	382.40	3.60	$[M+NH_4]^+$	ESI^+	400.12	215.10	14
					400.10	185.10	16
ZEN	318.36	6.91	$[M-H]^-$	ESI^-	317.11	175.03	25
					317.12	131.02	31
AOH	258.23	5.70	$[M+H]^+$	ESI^+	259.10	185.14	30
					259.11	128.13	49
TEA	197.23	3.32	$[M+H]^+$	ESI^+	198.12	139.12	14
					198.11	125.11	15
TEN	414.50	6.10	$[M+H]^+$	ESI^+	415.30	312.23	18
					415.31	256.23	25
FB_1	721.83	5.10	$[M+H]^+$	ESI^+	722.12	352.00	36
					722.11	334.00	45

T_R: retention time CE: collision energy

Table S5. The average daily intake of wheat and wheat-based products in Shanghai and participants for average body weight.

Area	Bw (kg)	Daily intake ($g·day^{-1}$)
Total population	58.20	83.37
Adult men	62.70	95.54
Adult women	54.00	72.17
7-10-year-old boys	38.30	98.65
7-10-year-old girls	33.40	75.13

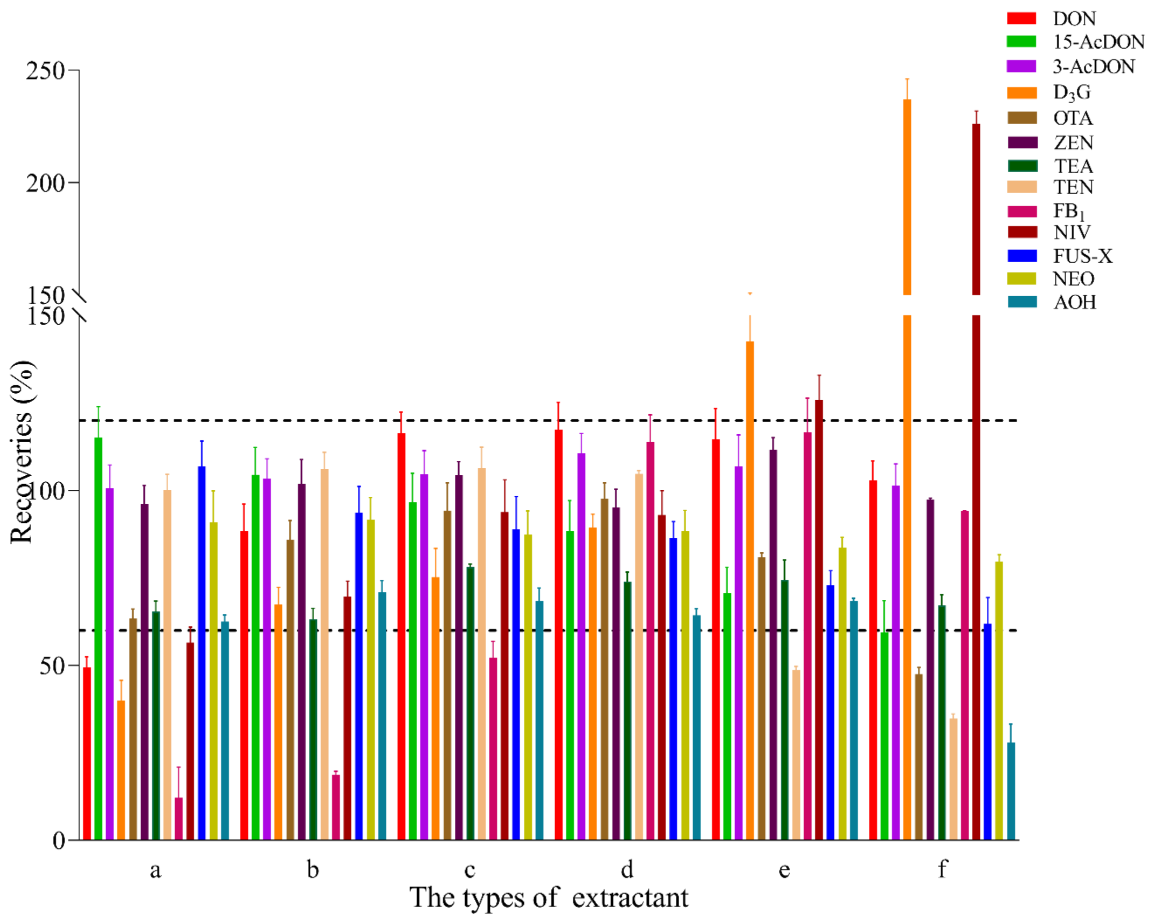

Figure S1. The effect of the extractant types containing 1% formic acid on the recoveries of mycotoxins: (a) 45% Acetonitrile, (b) 55% Acetonitrile, (c) 65% Acetonitrile, (d) 75% Acetonitrile, (e) 85% Acetonitrile, (f) 95% Acetonitrile. The two dashed lines represent recoveries of 60% and 120%, respectively.

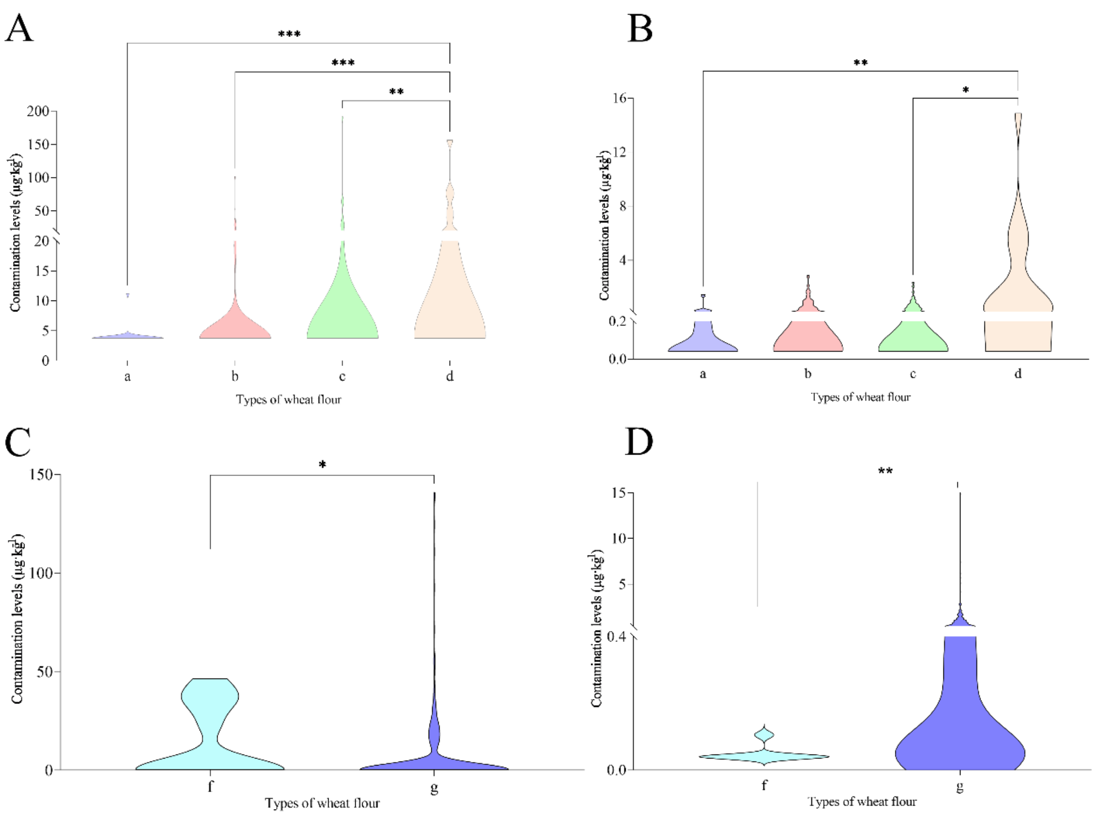

Figure S2. The distribution characteristics and differences of contamination levels for mycotoxins in various types of wheat flour samples: (A) occurrence of FUS-X in different wheat flour samples; (a) low-gluten wheat flours, (b) medium-gluten wheat flours, (c) high-gluten wheat flours, (d) whole wheat flours; (B) occurrence of TEN in different wheat flour samples; (a) low-gluten wheat flours, (b) medium-gluten wheat flours, (c) high-gluten wheat flours, (d) whole wheat flours; (C) occurrence of AOH in different wheat flour samples; (f) organic wheat flours, (g) conventional wheat flours; (D) occurrence of TEN in different wheat flour samples; (f) organic wheat flours, (g) conventional wheat flours.

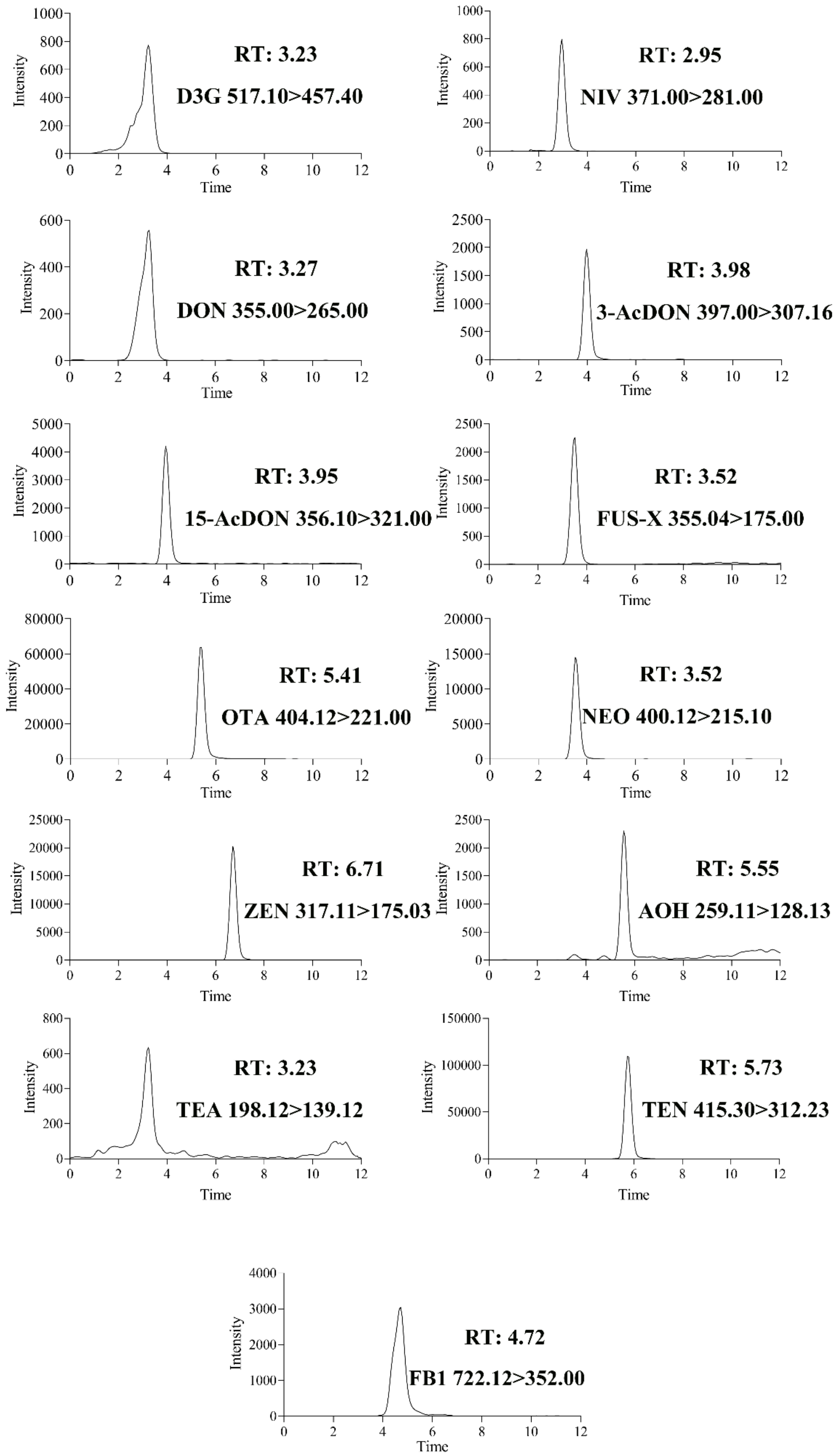

Figure S3. The chromatograms of 13 mycotoxins at middle concentration under optimized chromatographic and mass spectrometry conditions.
